# Embracing risk

**DOI:** 10.1242/dmm.021840

**Published:** 2015-08-01

**Authors:** Ross Cagan

**Affiliations:** Ross Cagan is Editor-in-Chief of *Disease Models & Mechanisms* and is Senior Associate Dean of the Graduate School of Biomedical Sciences at the Icahn School of Medicine at Mount Sinai, New York; Icahn School of Medicine at Mount Sinai, Annenberg 25-40, Campus Box 1020, 1468 Madison Avenue, New York, NY 10029, USA

**Keywords:** Risk, Entrepreneurship, Biomedicine

## Abstract

I entered the science field because I imagined that scientists were society's “professional risk takers”, that they like surfing out on the edge. I understood that a lot of science – perhaps even most science – has to be a solid exploration of partly understood phenomena. But any science that confronts a difficult problem has to start with risk. Most people are at least a bit suspicious of risk, and scientists such as myself are no exception. Recently, risk-taking has been under attack financially, but this Editorial is not about that. I am writing about the long view and the messages we send to our trainees. I am Senior Associate Dean of the graduate school at Mount Sinai and have had the privilege to discuss these issues with the next generation of scientists, for whom I care very deeply. Are we preparing you to embrace risk?

## Abstract (precis)

**Summary:** We are entering an era of unprecedented promise for solving some of humankind's most pressing health issues. In this Editorial, Ross Cagan emphasizes the importance of taking risks in science and in career choices.

## Culture of risk

As a long-standing academic scientist, I like my tenure and my funding to arrive with minimum risk. Although this is a reasonable response to wanting a good life, it is not always the optimal approach for solving 21st-century biomedical problems. It discourages risk-taking and encourages playing to the grant agencies. Worst of all, my generation's fear of risk bleeds down to you. We teach you how to design projects that are ‘bullet-proof’. You will build the Biomedicine Age, and you will need to take risks to do it.

“…a lot of science – perhaps even most science – has to be a solid exploration of partly understood phenomena. But any science that confronts a difficult problem has to start with risk”

A friend and serial entrepreneur once commented that “scientists can be quick to ‘go negative’…unusual ideas are met with ‘that won't work’”. I would not go that far, but he does have a point. Most risky ideas don't pan out and, if I just dismiss them, my batting average will be pretty high. But hard problems will never get solved, and some of the magic of science is lost. Try a lab meeting some time with a simple rule: no negative comments, only “yes, and…”. Perhaps the student's idea is not exactly right but the really great idea is lurking nearby. Kill the first, and the great idea is also lost. This all feeds to a culture of innovation and risk.

“Most risky ideas don't pan out and, if I just dismiss them, my batting average will be pretty high. But hard problems will never get solved, and some of the magic of science is lost”

## Risk in the Biomedicine Age

Biomedical science today is in a similar position as the computer industry of the early 1980s. Then, computer research was dominated by government funding and made up mainly by academics, mostly physicists and mathematicians. The government decided that it was not going to increase funding and the computer industry was faced with a challenge. The next generation was all in: they believed they could make a difference, and they did. They opened their garages and built the Information Age. If you tell someone in Silicon Valley that a great project is risky, that's considered a bonus.

We are now entering the Biomedicine Age. Our great challenge is to understand how our body works and bring that understanding to erasing disease. Cancer, heart disease, infection, neurodegenerative diseases and – perhaps our most profound health challenge – mental illness all represent hard problems that will transform who we are when they are solved. The key words here are ‘risk’ and ‘diversity’ (more on diversity in a future Editorial). Biomedical challenges will require biology PhD's entering basic research as explorers, industrial research to create therapeutics, Wall Street to build its financial underpinnings, and professional communicators to explain it all to society. The brains-in-the-garage are…you.

This will require loving risk. Start a company. It will probably fail (mine did), but then start another (working on that). Worried that funding for startups is tight, that you will confront the ‘valley of death’? There is a much better financial structure now in biomedicine than there was for computers 30 years ago. We need your creativity. Apps, small devices, computational algorithms, blogs, new materials, innovative teaching etc. are all part of the Biomedicine Age. Create a new way of thinking about old problems and find a way to make your solution happen. Then find someone who will mentor you, encourage you that your idea is actually great. Succeeding will be sweet.

